# Carbapenem-based versus non-carbapenem therapy for hospital-acquired and ventilator-associated pneumonia in a resource-limited setting: a retrospective cohort study of clinical outcomes

**DOI:** 10.3389/jpps.2026.17336

**Published:** 2026-09-28

**Authors:** Uroosa Kanwal, Iyad Naeem Muhammad, Ale Zehra, Syed Shaukat Ali Muttaqi Shah, Falak Abro, Tehreem Ansari, Najia Rahim, Rabya Munawar, Mujahid Hussain

**Affiliations:** 1 Department of Pharmacy Practice, Faculty of Pharmacy and Pharmaceutical Sciences, University of Karachi, Karachi, Pakistan; 2 Department of Pharmacy Practice, Faculty of Pharmacy, Salim Habib University, Karachi, Pakistan; 3 Department of Pharmacy Practice, Faculty of Pharmaceutical Sciences, Dow College of Pharmacy, Dow University of Health Sciences, Karachi, Pakistan; 4 Department of Clinical Pharmacy, College of Pharmacy, Al-Farahidi University, Baghdad, Iraq; 5 Department of Pediatrics, Liaquat National Hospital, Karachi, Pakistan; 6 Department of Infectious Diseases, Dow University Hospital, Dow University of Health Sciences, Karachi, Pakistan; 7 Department of Pharmaceutical Chemistry, Faculty of Pharmaceutical Sciences, Dow College of Pharmacy, Dow University of Health Sciences, Karachi, Pakistan; 8 Sindh Healthcare Commission, Karachi, Pakistan

**Keywords:** antimicrobial stewardship, carbapenem combination therapy, carbapenem monotherapy, hospital-acquired pneumonia (HAP), pathogen-specific mortality, ventilator-acquired pneumonia (VAP), carbapenem-based therapy

## Abstract

**Background:**

Hospital-acquired pneumonia (HAP) and ventilator-associated pneumonia (VAP) are major causes of in-hospital mortality and antimicrobial resistance (AMR), particularly in low- and middle-income countries (LMICs). Carbapenems are commonly used due to their broad-spectrum activity; however, their efficacy in real-world clinical practices comparing carbapenem with non-carbapenem regimens in these settings is limited.

**Objectives:**

To compare clinical outcomes between patients receiving carbapenem-based and non-carbapenem therapy for HAP and VAP at a tertiary care hospital in Pakistan.

**Methods:**

This retrospective cohort study included 93 adult patients (HAP: n = 62, VAP: n = 31) over the period of 1 year. Demographics, comorbidities, microbial isolates, treatment regimen, and clinical outcomes were analyzed using descriptive statistics, chi-square tests, and risk ratio calculations with 95% confidence interval. Multivariable logistic regression was planned to adjust for confounders but was not feasible due to missing severity score and biomarker data.

**Results:**

HAP was more common in females (63%), while VAP showed a male predominance (52%). The most common pathogens were *Acinetobacter spp.* (HAP: 11.3%; VAP: 19.4%) and *Pseudomonas aeruginosa* (HAP: 11.3%; VAP: 16.1%). The most prevalent comorbidity in HAP was diabetes mellitus (38.7%). Carbapenem-based therapy (meropenem) was used in 65.6% of cases and the most frequently prescribed non-carbapenem agents were piperacillin-tazobactam, ceftazidime, and colistin. No significant difference in mortality was observed between carbapenem and non-carbapenem therapy in HAP (31.6% vs. 29.2%; RR 1.08, 95% CI 0.50–2.36, p = 0.840) or VAP (65.2% vs. 50.0%; RR 1.30, 95% CI 0.61–2.77, p = 0.440). VAP was associated with significantly higher crude mortality than HAP (61.3% vs. 30.6%; p = 0.005). Crude mortality rates appeared highest among patients in the *Acinetobacter baumannii* group (70.4%) and *Pseudomonas aeruginosa* group (31.3%), though treatment group difference were not statistically significant.

**Conclusion:**

In this single-center retrospective cohort, no statistically significant association between carbapenem-based therapy and all-cause in-hospital mortality was detected in HAP or VAP. Crude mortality appeared highest among patients with *Acinetobacter* and *Pseudomonas* isolates. However, wide confidence intervals, treatment-selection bias, and unadjusted analyses preclude definitive conclusions. These findings should be considered hypothesis-generating, requiring confirmation in adequately powered, larger prospective studies.

## Introduction

Hospital-acquired pneumonia (HAP) refers to lung infections that develop 48 h or more of hospital admission, whereas ventilator-associated pneumonia (VAP) occurs ≥48 h following endotracheal intubation [1]. The primary cause of HAP and VAP in immunocompetent individuals is bacteria, while fungal and viral infections occur less frequently [2]. HAP and VAP remain among the most common healthcare-associated infections in developing countries, contributing a significant burden to increased morbidity, mortality, prolonged hospitalization, and healthcare costs [3].

In Asia, the prevalence of HAP remains high, representing a major burden in critical care settings [3–6]. A meta-analysis reported VAP rates of 18.5 per 1,000 ventilator-days in low- and middle-income countries (LMICs), more than twice those reported in high-income settings [6]. In Pakistan, data on HAP prevalence in general wards are scarce. Available studies by Imran et al. [4] and Kumar et al. primarily focus on intensive care units (ICUs), highlighting the under-recognized burden of HAP among non-ICU patients and the need for localized epidemiological data [4, 5].

Management of HAP and VAP is challenged by rising antimicrobial resistance (AMR) and high mortality, often associated with delayed or inadequate empirical therapy [7]. Empirical treatment is often initiated before microbiological results are available (typically within 48–72 h of diagnosis). Carbapenems such as meropenem and imipenem are frequently recommended for patients at high risk of multi-drug resistant infections, though guidelines emphasize that empirical choices be guided by local resistance patterns and individual risk factors [1, 8]. Other carbapenems, such as doripenem and ertapenem, are not recommended for HAP/VAP due to limited efficacy data or inappropriate spectrum of activity [8, 9]. However, increasing carbapenem resistance has raised concerns regarding overuse and the optimal choice between monotherapy and combination regimens, as guidelines recommend combination therapy for VAP and high risk HAP, and monotherapy to be considered for selected HAP cases [1, 8, 9].

Although carbapenem-based regimens have been evaluated for the treatment of HAP and VAP, evidence regarding mortality benefits remains mixed, and emerging resistance and limited regional data necessitate further investigation [9, 10]. Recent evidence suggests that carbapenems and newer β-lactam/β-lactamase inhibitor combinations including ceftazidime-avibactam and ceftolozane-tazobactam, have comparable outcomes, highlighting the necessity of personalized, pathogen-directed therapy [11].

This study addresses several important gaps in the literature. First, while carbapenems are widely used for HAP and VAP in resource-limited settings, real-world effectiveness data comparing monotherapy, combination therapy, and non-carbapenem regimens are limited. Second, most previous Pakistani studies have focused exclusively on ICU patients, leaving the burden of HAP in general wards largely unrecognized. Third, pathogen-specific mortality data in this population are limited. This study provides localized, real-world evidence to inform empiric antibiotic strategies and enhance stewardship initiatives in high-burden, resource-limited settings.

Based on the current evidence, we hypothesized that carbapenem-based therapy would not show a mortality benefit over non-carbapenem-based regimens in this setting, particularly given the high baseline severity of illness and the rising prevalence of carbapenem resistance. We also anticipated that pathogen-specific mortality would vary, with *Acinetobacter* and *Pseudomonas* associated with the poorest outcomes regardless of treatment choice.

## Materials and methods

### Study design and setting

The study was a retrospective cohort study that was conducted at the tertiary care private, non-profit hospital in Karachi, Pakistan. The sample size involved adult patients (at least 18 years old) admitted to the Coronary Care Unit (CCU), High Dependency Unit (HDU), the Intensive Care Unit (ICU), and the general wards who were diagnosed with the conditions hospital-acquired pneumonia (HAP) or ventilator-associated pneumonia (VAP).

### Study period and data collection

The research took place in a period of about 12 months, and data were collected by retrospective review of the patient’s medical records using non-probability purposive sampling from 1st January 2021 till 31st December 2021.

### Ethics statement

This study was approved by the Institutional Review Board (IRB) of IHHN, Pakistan (Approval reference: IHHN_IRB_2021_12_008). A formal exemption from full review was granted by IRB due to the retrospective nature of the study, as it involved only the analysis of de-identified patient data. All patient data were anonymized and de-identified prior to analysis. The requirement of informed consent was waived by the IRB. Patient confidentiality was maintained throughout the study in accordance with the Declaration of Helsinki.

### Eligibility

Inclusion criteria involved the patients who were 18 years old or above and had signs of pneumonia, International Classification of Diseases-10 (ICD-10) codes (J13-J18, J85). The exclusion criteria were patients who were immunosuppressed or on chemotherapy, pregnant women, and patients transferred from another hospital who developed pneumonia within 48 h of transfer.

### Study tool

A clinical checklist (Supplementary Material 1), which includes the demographic data, diagnostic data, comorbidities, laboratory results, and therapeutic regimens that comprise carbapenem monotherapy or combination therapy, as well as the identified drug-related issues, was developed by us in the aftermath of a complete literature review [4, 5, 8–11]. The checklist was evaluated by a panel of experts consisting of infectious disease experts and board-certified pharmacotherapy specialists (BCPS). After the review of the patient’s medical records, cases were recognized as HAP or VAP according to the definitions provided by the American Thoracic Society (ATS) and the Infectious Diseases Society of America (IDSA) [2].

### Data collection process

The sample size was determined by all eligible cases during the 12-month study period, with no *a priori* power calculation. Out of the 24,565 patients that were admitted in the 12 months study period, 93 qualified as per the inclusion criteria of the study. Patients having HAP and VAP were identified using ICD-10 codes (J13-J18, J85). All admissions during the study period were electronically screened using ICD-10 codes to identify potential pneumonia cases. The medical records of all identified pneumonia cases were reviewed independently by two investigators, a board-certified infectious disease pharmacist and an infectious disease (ID) physician, to confirm the diagnosis of HAP or VAP using ATS/IDSA definitions. Reviewers were not blinded to treatment or outcomes, as these were part of clinical record. Disagreements were resolved by consultation, with a third ID specialist consulted if consensus could not be reached. Each patient contributed only one episode, in case of multiple pneumonia related episodes during the enrollment period, only the first episode of pneumonia was included. The time of admission, endotracheal intubation, symptom onset, and imaging was recorded for each case using electronic medical records and clinical notes. The checklist was used to analyze the profile of each patient to assess the carbapenem efficacy by analyzing the symptom resolution, pathogen isolation in the culture, the length of therapy, and the mortality or the survival rates [12, 13].

### Definitions

Pneumonia Type: Hospital-acquired pneumonia (HAP): Pneumonia developing ≥48 h after hospital admission, excluding patients on mechanical ventilation. Ventilator-associated pneumonia (VAP): Pneumonia developing ≥48 h after endotracheal intubation.

Diagnosis of HAP and VAP needed presence of a new or growing infiltrate on imaging (chest X-ray or CT scan) along with presence of at least two of the following clinical criteria: fever (temperature greater than 38 °C or less than 36 °C); leukocytosis (>12,000 cells/mm^3^) or leukopenia (<4,000 cells/mm^3^); worsening of oxygenation (PaO_2_/FiO_2_ ratio decline); or purulent respiratory secretions.

In order to differentiate HAP from community-acquired pneumonia (CAP), patients who were presented with pneumonia on admission or developed pneumonia in the first 48 h of hospitalization were excluded. Patients with onset of pneumonia ≥48 h after admission were classified as HAP, following ATS/IDSA definitions.

Treatment Groups: Carbapenem therapy: Patients receiving meropenem as part of their antibiotic regimen. During the study period, although imipenem was available in the hospital formulary, but was not prescribed for any patient with HAP or VAP, likely due to local prescribing habits and microbial stewardship program. Meropenem was administered at a standard adult dose of 1 g every 8 hourly as a 30-min intravenous infusion, with renal dose adjustments as per the hospital guidelines (creatinine clearance <50 mL/min - 1 g every 12 h; <30 mL/min - 500 mg every 12 h; <10 mL/min - 500 mg every 24 h). The duration of therapy was determined by the treating physician based on clinical response and microbiological findings. Non-carbapenem therapy: Patients receiving antibiotic regimens without any carbapenem. The doses of non-carbapenem regimens (piperacillin-tazobactam, ceftazidime, and colistin) were administered as per standard hospital protocols, with dosing based on renal function and body weight. A summary of all treatment regimens is provided in Supplementary Table S1.

Classification of treatment exposure was based on the initial empirical antibiotic regimen initiated within 48 h of HAP/VAP diagnosis. To be included in the treatment group patients were required to have received at least 48 h of the assigned therapy. Patients who were prescribed both carbapenem and non-carbapenem regimens sequentially, were classified based on their initial empirical regimen (intention-to-treat principle) during the hospital stay. The changes in the treatment (switches, escalations, or de-escalations) were recorded but did not alter the initial group assignment.

Critical illness was defined as patients requiring admission to the ICU or MICU with evidence of organ dysfunction, mechanical ventilation, or vasopressor support. Non-critical patients were those admitted to the HDU, CCU, or general wards without the need for mechanical ventilation or vasopressor support.

Pathogen Groups (for mortality analysis): For mortality analysis, pathogen groups were created using a hierarchical, mutually exclusive classification. Polymicrobial infections were assigned to the most clinically significant pathogen based on the following priority: (1) *Acinetobacter baumannii, (2) Pseudomonas aeruginosa,* and (3) other pathogens. Patients with no organism isolated were classified as “No growth”. *Acinetobacter baumannii* group: Isolates of *A. baumannii* alone or in polymicrobial infections (priority 1). *Pseudomonas aeruginosa* group: Isolates of *P. aeruginosa* alone or in polymicrobial infections, excluding those already assigned to the *Acinetobacter* group (priority 2). Other pathogens group: Isolates including *Klebsiella pneumoniae, Escherichia coli, Staphylococcus aureus* (including MRSA), *Enterobacter* species, or other polymicrobial combinations not containing *Acinetobacter* or *Pseudomonas* (priority 3). No growth: Negative culture results.

### Statistical analysis

Statistical analysis was performed using Statistical Package of the Social Sciences (SPSS) version 26 (IBM Inc., Chicago, IL, USA). The descriptive statistics were used to describe demographic features of patients, clinical and microbiological characteristics. Continuous variables are presented as mean (± standard deviation) or median (interquartile range, IQR) as appropriate. Categorical variables are presented as frequencies and percentages.

The primary outcome was all-cause in-hospital mortality. Unadjusted risk ratios (RR) with 95% confidence intervals (CI) were calculated to compare mortality between carbapenem and non-carbapenem therapy. Chi-square or Fisher’s exact tests were used for categorical comparisons, as appropriate.

Pre-specified subgroup analyses were conducted to evaluate the consistency of treatment effects. Subgroups were defined by: critical illness status - critical vs. non-critical; ICU admission: ICU (MICU and ICU) vs. non-ICU (HDU, CCU, and general ward); Diabetes status (‘Comorbidities’): diabetic (DM) vs. non-diabetic (other or no comorbidity). For each subgroup, RR and 95% CI were calculated using cross-tabulation with the risk estimate function. A two-tailed p-value <0.05 was considered statistically significant. Due to small sample sizes in some subgroups, results were interpreted with caution, as the diabetic subgroup analysis included 30 patients (23 carbapenem-treated and 7 non-carbapenem-treated).

The secondary outcome analysis included hospital stay compared between treatment groups using the Mann-Whitney U test. Median hospital stay categories with IQR are reported.

Microbiological isolates were categorized into mutually exclusive pathogen groups based on a hierarchical classification (see definitions). Mortality rates were calculated for each pathogen group overall and stratified by pneumonia type (HAP vs. VAP) and treatment group (carbapenem vs. non-carbapenem). Pathogen-specific mortality analysis was performed to compare mortality across pathogen groups and between treatment groups. For each pathogen group, mortality rates were compared between carbapenem and non-carbapenem therapy using Fisher’s exact test (due to small expected cell counts). Risk ratios (RR) with 95% CI were calculated for each pathogen group.

Biomarker levels (WBC, neutrophils, CRP, procalcitonin, temperature, and creatinine) were compared between baseline (diagnosis) and death in deceased patients using paired t-tests. Data are presented as median (interquartile range, IQR). A two-tailed p-value <0.05 was considered statistically significant. A multivariable binary logistic regression model was planned to adjust for age, diabetes, and pneumonia type. However, severity scores (SOFA, APACHE II) and biomarker levels were not available in the retrospective dataset; therefore, the adjusted analysis was not feasible, and results are presented as unadjusted estimates only. The wide confidence intervals in the subgroup and pathogen-specific analyses may reflect the small sample size, therefore, absence of statistically significant differences to be interpreted as inconclusive and not as an evidence of equivalence. All key variables reported in this study (age, gender, comorbidities, pneumonia type, critical illness, ward, LOS, pathogen, treatment, and mortality) had complete data. All tests were two-tailed, and p-values <0.05 were considered statistically significant. Detailed subgroup analyses and pathogen-specific mortality stratified by pneumonia type and treatment group are provided in Supplementary Table S2 and Supplementary Table S3.

Figure 1 presents a flowchart illustrating patient enrollment, exclusion criteria, study groups, and the statistical analysis performed.

**FIGURE 1:**
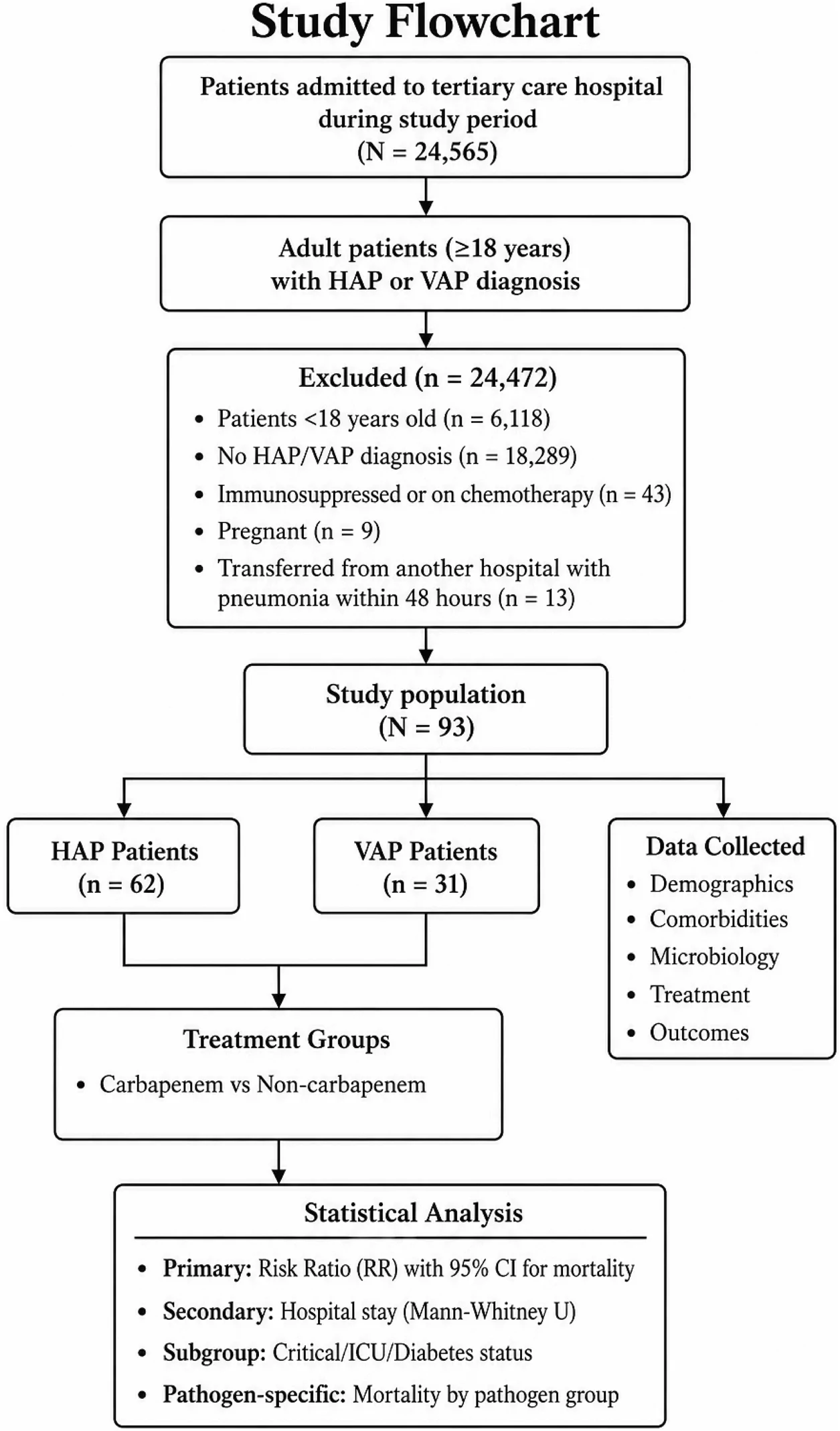
Study flowchart. Flowchart illustrating patient enrollment, exclusion criteria, study groups, and statistical analyses. A total of 24,565 patients were screened, of whom 93 met the inclusion criteria for HAP or VAP. Patients were classified into carbapenem-based and non-carbapenem-based treatment groups. Statistical analysis included unadjusted risk ratios for mortality, hospital stay comparisons, subgroup analyses, and pathogen-specific mortality.

## Results

### Patient characteristics

Table 1 presents the demographic and clinical characteristics of patients with HAP (n = 62, 66.7%) and VAP (n = 31, 33.3%). Most of the patients (HAP: 48; VAP: 23) were adults, while the remainder (HAP: 14; VAP: 8) were geriatrics (>65 years). HAP was more common in females (63%), whereas VAP was slightly higher in males (52%). Additionally, HAP cases predominated in the general ward (40.3%) and HDU (22.6%), while VAP was more common in the ICU (74.2%) and CCU (25.8%; p < 0.001), showing a significant association between pneumonia type (HAP and VAP) and ward location (*p <* 0.001). Among HAP patients, (n = 54, 87.1%) reported no smoking or illicit drug use. Similarly, (n = 27, 87.1%) of VAP had no such habits, while (n = 4, 12.9%) were smokers. The difference between groups was not statistically significant (*p =* 0.54). Fever with respiratory complaints were the most frequent presenting symptoms in both groups, but were significantly more frequent in VAP (90.03% vs. 66.1%; p = 0.012). Diabetes mellitus was the most prevalent comorbidity among HAP patients (38.7%), while other co-morbidities (hypertension, chronic kidney disease, COPD) were more frequent in VAP patients (48.4%), though the overall co-morbidity distribution did not differ significantly between groups (p = 0.060).

**TABLE 1:** Demographic, clinical, and comorbid characteristics of patients with HAP and VAP.

Parameter	HAP (n = 62) n (%)	VAP (n = 31) n (%)	P-Value
Age groups	​		0.748
Adult (18–65 years)	48 (77.4)	23 (74.2)	
Geriatric (>65 years)	14 (22.6)	8 (25.8)	
Gender distribution	​		0.181
Male	23 (37.1)	16 (51.6)	
Female	39 (62.9)	15 (48.4)	
Ward distribution	​		<0.001
ICU	18 (29.0)	23 (74.2)	
CCU	5 (8.1)	8 (25.8)	
HDU	14 (22.6)	0 (0)	
General ward	25 (40.3)	0 (0)	
Habits/Addictions	​		0.549*
Smokers	6 (9.7)	4 (12.9)	
Illicit drug users	2 (3.2)	0 (0)	
No smoking/illicit drug use	54 (87.1)	27 (87.1)	
Presenting complaints	​		0.012
Fever with respiratory complaints	41 (66.1)	28 (90.3)	
Other complaints	21 (33.9)	3 (9.7)	
Co-morbidity	​		0.060
Diabetes mellitus	24 (38.7)	6 (19.4)	
Other than diabetes mellitus**	16 (25.8)	15 (48.4)	
No co-morbidity	22 (35.5)	10 (32.3)	

* Fischer’s exact test used due to small expected cell counts.

** Other comorbidities included hypertension, chronic kidney disease, and chronic obstructive pulmonary diseases. Abbreviations: HAP, hospital-acquired pneumonia; VAP, ventilator-associated pneumonia; ICU, intensive care unit; CCU, coronary care unit; HDU, high dependency unit.

### Baseline characteristics by treatment group

Baseline characteristics by treatment group are presented in Supplementary Table S4. The carbapenem and non-carbapenem groups were comparable in terms of age (mean 52.21 vs. 50.28 years; p = 0.668) and gender distribution (p = 0.853). However, carbapenem-treated patients had a higher proportion of diabetes (37.7% vs. 21.9%), VAP (37.7% vs. 25.0%), and critical illness (77.0% vs. 62.5%), though these differences did not reach statistical significance.

### Microbiological findings

Table 2 summarizes the microbiological isolates from HAP and VAP patients. Gram-negative bacteria predominated in both groups. The most common pathogens were *Acinetobacter baumannii (HAP: 11.3%; VAP: 19.4%) and Pseudomonas aeruginosa (HAP: 11.3%; VAP: 16.1%).* Polymicrobial infections were significantly more common in VAP (41.9%) than in HAP (8.1%). Notably, culture negative pneumonia was frequent in HAP (45.2%) but uncommon in VAP (9.7%). A detailed breakdown of polymicrobial isolates is provided in Supplementary Table S5.

**TABLE 2:** Microbiological Isolates in HAP and VAP patients.

Types of pathogens	HAP (n = 62) %	VAP (n = 31) %
Gram-negative bacteria		
*Acinetobacter baumannii*	7 (11.3)	6 (19.4)
*Pseudomonas aeruginosa*	7 (11.3)	5 (16.12)
*Klebsiella pneumoniae*	3 (4.83)	2 (6.4)
*Escherichia coli*	5 (8.06)	0
*Enterobacter*	0	1 (3.22)
Gram-positive bacteria		
*Methicillin-resistant Staphylococcus aureus*	2 (3.22)	0
*Staphylococcus aureus*	1 (1.6)	1 (3.22)
Multiple gram-negative bacteria	​	​
*Acinetobacter baumannii+ Pseudomonas aeruginosa*	1 (1.6)	3 (9.67)
*Acinetobacter baumannii+ Klebsiella pneumoniae*	0	2 (6.4)
*Acinetobacter baumannii+ Pseudomonas aeruginosa+* *Klebsiella pneumoniae*	0	1 (3.22)
Combination (gram-positive + negative)		
*Acinetobacter baumannii+ Staphylococcus aureus*	2 (3.22)	2 (6.4)
*Pseudomonas aeruginosa+Methicillin-resistant Staphylococcus aureus+ Escherichia coli*	1 (1.6)	0
*Pseudomonas aeruginosa+Staphylococcus aureus*	1 (1.6)	1 (3.22)
*Acinetobacter baumannii+ Pseudomonas aeruginosa+* *Staphylococcus aureus*	0	2 (6.4)
*Acinetobacter baumannii+ Klebsiella pneumoniae+ Staphylococcus aureus*	0	1 (3.22)
*Pseudomonas aeruginosa + S. pneunomiae + H Influenzae*	0	1 (3.22)
*Others: Candida Spp.*	4 (6.45)	0
*Not isolated*	28 (45.2)	3 (9.67)

Abbreviations: HAP, hospital-acquired pneumonia; VAP, ventilator-associated pneumonia.

### Biomarker trends in deceased patients

Among the 93 patients included in the study, mortality was 30.6% (19/62) for HAP and 61.3% (19/31) for VAP. Changes in laboratory parameters from diagnosis to death were evaluated in these 38 deceased patients (Figure 2). In HAP patients, median WBC increased significantly from 13.1 (IQR 8.2–21.6) at baseline to 17.4 (IQR 13.3–25.8) at death (p = 0.036). In VAP patients, both WBC (14.55–18.3; p = 0.030) and neutrophils (88.4%–91.5%; p = 0.001) increased significantly. CRP, procalcitonin, temperature, and creatinine showed no significant changes in this exploratory analysis, likely due to small sample sizes and wide-individual variability. These exploratory findings should be interpreted with caution.

**FIGURE 2:**
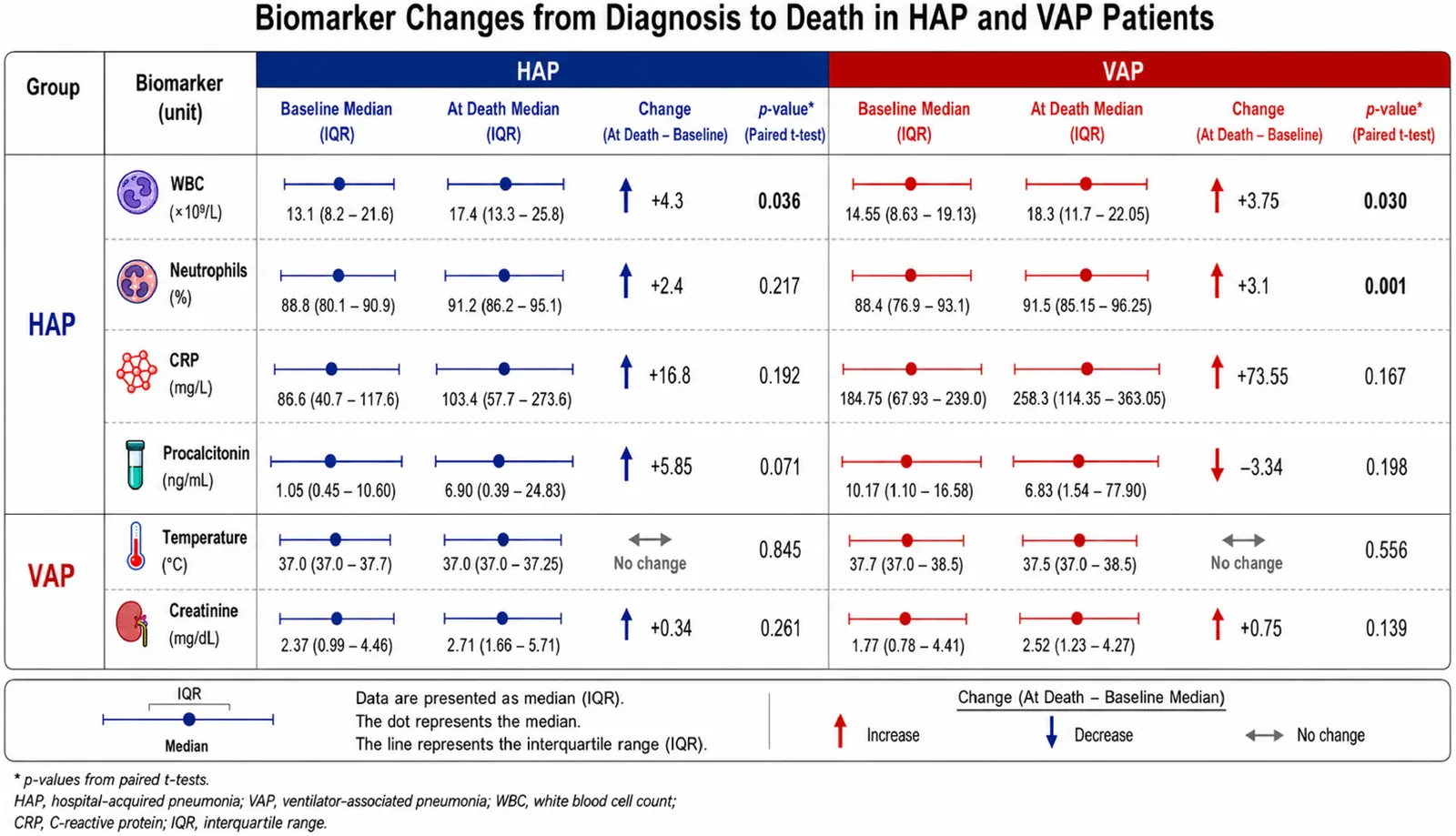
Biomarker changes from diagnosis to death in HAP and VAP patients. Changes in white blood cell count (WBC) and neutrophils from baseline (diagnosis) to death in deceased patients (HAP: n = 19; VAP: n = 19). Data are presented as median values with interquartile ranges (IQR). (A) HAP patients: WBC increased from 13.1 (IQR 8.2–21.6) at baseline to 17.4 (IQR 13.3–25.8) at death (p = 0.036). (B) VAP patients: WBC increased from 14.55 to 18.3 (p = 0.030) and neutrophils from 88.4% to 91.5% (p = 0.001). This analysis is exploratory due to the small sample size.

### Analysis of carbapenem monotherapy, combination regimen with and without carbapenem

Therapeutic approaches for HAP patients (n = 62) showed distinct patterns. Carbapenem-based regimens were administered to 38 (61.3%) of patients, While non-carbapenem alternatives were used in 24 (38.7%) of cases. Among 31 VAP patients, 23 (74.2%) received carbapenem-based therapy, while 8 (25.8%) were treated with non-carbapenem regimens. Meropenem was consistently prescribed in all carbapenem-based treatments. In the non-carbapenem group, piperacillin-tazobactam was the most frequently prescribed beta-lactam agent for HAP patients. However, piperacillin-tazobactam, ceftazidime, and colistin were prescribed with equal frequency in VAP cases.

### Clinical outcomes of treatment

Table 3 shows that no statistically significant differences were observed in mortality or hospital stay between carbapenem and non-carbapenem therapy for HAP, VAP, or the combined cohort (all p > 0.05).

**TABLE 3:** Clinical outcomes by treatment regimen in HAP and VAP patients.

Outcome	Non-carbapenem therapy	Carbapenem therapy	p-value
**HAP (n = 62)**	n = 24	n = 38	​
Mortality, n (%)	7 (29.2)	12 (31.6)	0.840
Unadjusted RR for death (95% CI)	Reference	1.08 (0.50–2.36)	​
Median hospital stay (IQR)	8 (3–17)	8 (4–13)	0.867
**VAP (n = 31)**	n = 8	n = 23	​
Mortality, n (%)	4 (50.0)	15 (65.2)	0.440
Unadjusted RR for death (95% CI)	Reference	1.30 (0.61–2.77)	​
Median hospital stay (IQR)	14 (9–36)	13 (7–19)	0.214
**Combined HAP + VAP (n = 93)**	n = 32	n = 61	​
Mortality, n (%)	11 (34.4)	27 (44.3)	0.355
Unadjusted RR for death (95% CI)	Reference	1.29 (0.74–2.24)	​
Combined hospital stay (IQR)	10 (6–17)	9 (5–14)	0.767

Abbreviations: HAP, hospital-acquired pneumonia; VAP, ventilator-associated pneumonia; RR, risk ratio; CI, confidence interval; IQR, interquartile range.

Among 62 HAP patients, mortality was 31.6% in the carbapenem group (12/38) versus 29.2% in the non-carbapenem group (7/24). The unadjusted risk ratio for death with carbapenem was 1.08 (95% CI: 0.50–2.36; p = 0.840). In the carbapenem group median length of stay (LOS) was 8 days (IQR 4–13), and in the non-carbapenem group was 8 days (IQR 3–17) p = 0.867. For VAP patients, median LOS was 13 days (IQR 7–19) in the carbapenem group versus 14 days (IQR 9–36) in the non-carbapenem group (p = 0.214). The difference observed in the combined cohort, were not statistically significant (p = 0.767). Supplementary Table S6, shows the comparison of LOS between survivors and non-survivors, median LOS was 7 days (IQR 3–13) for HAP patients who died versus 8 days (IQR 5–15) for those who survived (p = 0.520). In VAP patients, median LOS was 10 days (IQR 4–18) for non-survivors versus 19.5 days (IQR 10.5–30.5) for survivors (p = 0.109). Although not statistically significant, the longer LOS in VAP survivors likely reflects prolonged ICU and mechanical ventilation requirements.

Among 31 VAP patients, mortality was 65.2% in the carbapenem group (15/23) versus 50.0% in the non-carbapenem group (4/8). The unadjusted risk ratio for death with carbapenem therapy was 1.30 (95% CI: 0.61–2.77; p = 0.440).

Subgroup analysis by critical illness status and ICU admissions did not reveal any significant differences in mortality between carbapenem and non-carbapenem therapy. In critical patients, the risk ratio for death with carbapenem therapy was 1.11 (95% CI 0.67–1.84; p = 0.689); in ICU patients, the risk ratio was 1.33 (95% CI 0.76–2.31; p = 0.269), and no significant mortality benefit was observed in diabetic (RR 1.01, 95% CI 0.38–2.69, p = 1.000) or non-diabetic patients (RR 1.40, 95% CI 0.71–2.74, p = 0.431), as shown in Supplementary Table S2.

Overall, carbapenem-based therapy was not associated with significantly improved survival or shorter hospital stay compared to non-carbapenem regimens in either HAP or VAP.

### Pathogen-specific mortality by treatment

Table 4 presents mortality rates stratified by mutually exclusive pathogen groups and treatment type. The *Acinetobacter* group had the highest overall mortality (70.4%), with 77.8% mortality in carbapenem-treated patients versus 55.6% in non-carbapenem-treated patients (RR 1.40, 95% CI 0.74–2.64, p = 0.264). The *Pseudomonas* group had an overall mortality of 31.3% (50.0% vs. 12.5%; RR 4.00, 95% CI 0.56–28.4, p = 0.282). Other pathogens had the lowest overall mortality (21.1%), while no growth cases had intermediate mortality (32.3%). The differences between treatment groups for any pathogen group were not statistically significant. The wide confidence intervals reflect small sample sizes, particularly in the non-carbapenem subgroups for *Acinetobacter* (n = 9) and *Pseudomonas* (n = 8), should be interpreted as exploratory rather than definitive.

**TABLE 4:** Mortality by pathogen group and treatment.

Pathogen group	Total (n)	Carbapenem (n/N, %)	Non-carbapenem (n/N, %)	RR (95% CI)	p-value*
*Acinetobacter* group	27	14/18 (77.8)	5/9 (55.6)	1.40 (0.74–2.64)	0.264
*Pseudomonas* group	16	4/8 (50.0)	1/8 (12.5)	4.00 (0.56–28.4)	0.282
Other pathogens	19	3/14 (21.4)	1/5 (20.0)	1.07 (0.14–8.17)	1.000
No growth	31	6/21 (28.6)	4/10 (40.0)	0.71 (0.26–1.96)	0.686

Abbreviations: RR, risk ratio; CI, confidence interval.

* Fisher’s exact test used due to small expected cell counts.

Pathogen groups are mutually exclusive. Polymicrobial infections were assigned using a hierarchy: *Acinetobacter* baumannii > *Pseudomonas aeruginosa* > other pathogens. Patients with no growth are classified separately.

## Discussion

In this cohort, HAP was more frequently observed in females (63%), while VAP showed a male predominance (52%), though this gender difference was not statistically significant (p = 0.181), while previous studies had been reported HAP is more common in males [4, 5]. While previous Pakistani studies focused exclusively on ICU patients, we found that VAP cases were high in the ICUs (74%), and 40.3% of HAP cases originated in general wards (Table 1). This finding highlights the need for institution-wide prevention of infections [14]. Diabetes mellitus was the most common comorbidity in the HAP patients (Table 1), which is similar to the reported studies showing an increased prevalence of diabetes after respiratory infections in HAP and VAP. In VAP cases, however, our results are opposite to this observation [15], but rather similar to the results of another study that did not find any significant correlation between diabetes mellitus and VAP [16], consistent with previous reported studies.

Microbiological results showed that the common pathogens of HAP and VAP were Gram-negative. The most commonly reported pathogens were *Acinetobacter baumannii, Pseudomonas aeruginosa,* and *Klebsiella pneumoniae* (Table 2), which aligns with the global surveillance statistics showing that these pathogens are major causes of nosocomial pneumonia [5, 17, 18]. Polymicrobial infections were significantly more common in VAP (41.9%) than in HAP (8.1%), while culture negative pneumonia was frequent in HAP (45.2%) but uncommon in VAP (9.7%). A detailed breakdown of polymicrobial isolates is provided in Table 2. These organisms are able to survive in hospital settings and become multidrug resistant, causing treatment failure and deaths [19–21]. This highlights the necessity of efficient antibiotic stewardship initiatives, early pathogen detection, and rigorous infection control strategies to enhance patient outcomes [22, 23].

In this study, carbapenem-based therapy was not associated with significantly improved survival compared to non-carbapenem regimens in HAP, VAP, or the combined cohort (Table 3). For HAP patients, mortality was comparable between treatment groups, and the risk of death was not significantly different with carbapenem use. Hospital stay durations also did not differ significantly between treatment groups.

Crude mortality was higher among VAP patients (61.3% vs. 30.6%, χ^2^ = 8.03, p = 0.005), although this comparison may reflect substantial differences in illness severity, prolonged ventilation, care location, and other unmeasured factors [24]. It has been documented that the VAP-attributable mortality is estimated at 48%, with higher rates in critically ill and surgical ICU patients [25]. The findings highlight the importance of timely diagnosis and enhanced infection prevention measures [26].

The finding of no mortality benefit with carbapenem therapy must be interpreted in light of baseline imbalances between treatment groups (Supplementary Table S4). At baseline carbapenem-treated patients were sicker, and had higher proportions of critical illness, VAP, and diabetes. This confounding by indication may have affected any potential benefit of carbapenems.

Although combination therapy was more commonly used than monotherapy in VAP patients, the small number of VAP survivors (n = 12) precludes any meaningful comparison of treatment specific outcomes [27]. Therefore the formal comparison between monotherapy and combination was not performed. The LOS findings (Table 3) should be interpreted with caution, as the competing risk of death may confound association between treatment and hospital duration [28, 29].

A key finding in this study is the variation in crude mortality by pathogen group (Table 4). The *Acinetobacter* group was associated with the highest observed mortality (70.4% overall), followed by the *Pseudomonas* group (31.3%). Even among carbapenem-treated patients, mortality remained high for the *Acinetobacter* group (77.8%), with a borderline non-significant difference compared to non-carbapenem-treated patients (55.6%; RR 1.40, 95% CI 0.74–2.64, p = 0.264). The *Pseudomonas* group showed a similar pattern (50.0% vs. 12.5%; RR 4.00, 95% CI 0.56–28.4, p = 0.282). The observed mortality differences may reflect organism characteristics, antimicrobial resistance, treatment appropriateness, illness severity, host factors, or residual confounding. The present analysis cannot distinguish among these explanations. Although the biomarker analysis was exploratory, the observed trends in WBC and neutrophil increase in deceased patients are consistent with progressive infection and systemic inflammation, however, these findings require validation in larger prospective studies. Among deceased patients, WBC increased significantly in both HAP (p = 0.036) and VAP (p = 0.030), while neutrophils increased significantly only in VAP (p = 0.001), reflecting progressive infection and systemic inflammation as patients deteriorated towards death. The more pronounced inflammatory response in VAP patients may be associated to prolonged mechanical ventilation and ICU-related stress, which increases systemic inflammation. Other biomarkers showed non-significant trends, likely due to small sample sizes and wide inter-individual variability. Subgroup analyses by critical illness status (Supplementary Table S2) showed no significant mortality benefit of carbapenem therapy in any subgroup. In critical patients, the risk ratio for death with carbapenem therapy was 1.11 (95% CI 0.67–1.84; p = 0.689), and in ICU patients, the risk ratio was 1.33 (95% CI 0.76–2.31; p = 0.269). However, the wide confidence intervals in these subgroup analysis preclude definitive conclusion.

The findings of no mortality benefit with carbapenem therapy are consistent with reported studies. Combination therapy of carbapenem was not associated with reduced mortality in Gram-negative HAP/VAP [13]. Similarly, a meta-analysis by Howatt et al. [9] reported no significant advantage of carbapenems over other β-lactams in VAP [9].

A clinically meaningful mortality reduction in HAP/VAP would typically be in the range of 5–10% absolute risk reduction [9]. The wide confidence intervals observed in this study (e.g., RR 1.08, 95% CI 0.50–2.36 for HAP; RR 1.30, 95% CI 0.61–2.77 for VAP) cannot exclude such a difference. Therefore, while no statistically significant benefit was detected, the study does not rule out a clinically meaningful benefit or harm of carbapenem therapy. This underscores the need for larger, adequately powered studies to provide more precise estimates.

This study has several limitations. First, the retrospective, single-center design limits generalizability. Second, the small sample size and small subgroups resulted in wide confidence intervals indicate that the study was underpowered to detect clinically meaningful differences; therefore, the absence of statistically significant differences should be interpreted as inconclusive rather than evidence of equivalence. Third, confounding by indication is a major limitation, carabepenem-treated patients were sicker at baseline, and the analysis was unadjusted, precluding estimation of causal treatment effect. Fourth, diagnostic and ICD-code misclassification is possible, as pneumonia diagnosis relied on administrative codes and retrospective chart review. Fifth, exposure misclassification may have occurred after treatment switching, as patients who received both carbapenem and non-carbapenem regimens sequentially were classified based on their initial empirical regimen. Sixth, the lack of treatment-timing data limited our ability to assess the impact of delayed therapy. Seventh, the outcome was all-cause in-hospital mortality, not pneumonia-attributable mortality, which may overestimate the effect of pneumonia on death. Eight, LOS analysis was limited by the competing risk of death, as patients who died earlier may have shorter hospital stays despite worse outcomes, especially in VAP patients, where survivors had LOS than non-survivors (19.5 vs. 10 days), although this difference was not statistically significant (p = 0.109). Ninth, pathogen groups were classified using a mutually exclusive hierarchy to address overlapping polymicrobial infections, and the small number of polymicrobial cases limited the ability to perform more detailed subgroup analyses. Tenth, absence of severity scores (e.g., SOFA, APACHE II) limited the ability to adjust for illness severity, and the analysis was exploratory due to limited number of deceased patients, these findings should be interpreted with caution and are hypothesis-generating rather than definitive. Despite these limitations, this study provides important real-world data from a resource-limited setting.

In this single-center retrospective cohort, carbapenem-based therapy was not associated with a statistically significant survival benefit compared to non-carbapenem regimens in HAP or VAP. Crude mortality appeared highest among patients with *Acinetobacter baumannii* and *Pseudomonas aeruginosa* isolates. However, due to the small sample size, lack of adjustment for confounding, and retrospective design, these findings should be considered hypothesis-generating rather than definitive. The results highlight the need for personalized antibiotic selection based on local resistance patterns, susceptibility of pathogens, and disease severity, but requires confirmation in larger, adequately powered prospective studies.

## Data Availability

The original contributions presented in the study are included in the article/Supplementary Material, further inquiries can be directed to the corresponding authors.
