# Supplementary material for: Carbapenem-based versus non-carbapenem therapy for hospital-acquired and ventilator-associated pneumonia in a resource-limited setting: a retrospective cohort study of clinical outcomes
Source: J Pharm Pharm Sci. 2026 Sep 28;29:17336. doi: 10.3389/jpps.2026.17336 (PMC13654855; doi:10.3389/jpps.2026.17336)
Supplement: Supplementary file 1 [file DataSheet1.docx]

Form#001

Appendix I

**Patient Demographic Information**

Patient MR/ID or Case No:

Gender

Male	female

Age

18 – 27 years	Unit/Ward

28 – 37 years	MICU

38 – 47 years	ICU

48 – 57 years	HDU

58 – 67 years	Ward

68 – 77 years

78 – 87 years

88 – 97 years

98 years and above

Social Life Style

Smoker	Alcoholic	Illicit Drug user

**Patient Diagnostic and comorbidities information**

| Diagnosis  HAP  VAP  Culture Source  Blood Sputum  Urine Pleural fluid  Pus | Comorbidities  Diabetes CKD  Asthma Anemia  COPD HTN  CLD CNS disorder  Ulcer Auto immune  IHD TB  CVS | Microorganism in culture  Acinetobacter Baumannii  _Pseudomonas Aeruginosa  _Streptococcus Pneumoniae  _Klebsiella Pneumoniae  _ Staphylococcus Aureus  _Moraxella catarrhalis  _Haemophilus influenzae  _MRSA  _Candida.  _ Aspergillosis  _Others:_____________________ |
| --- | --- | --- |

**HOPI:**

Fever

Cough

Shortness of breath

**Laboratory Information:**

| **HAEMATOLOGY** | Normal | Day 1 | Day 2 | Day 3 | Day 4 | Day 5 | Day 6 | Day  7 | Day 8 | Day 9 | Day 10 | Day  11 | Day  12 | Day 13 | Day  14 |
| --- | --- | --- | --- | --- | --- | --- | --- | --- | --- | --- | --- | --- | --- | --- | --- |
| WBCs |  |  |  |  |  |  |  |  |  |  |  |  |  |  |  |
| Neutrophils |  |  |  |  |  |  |  |  |  |  |  |  |  |  |  |
| **INFLAMMATORY**  **MARKERS** |  |  |  |  |  |  |  |  |  |  |  |  |  |  |  |
| CRP |  |  |  |  |  |  |  |  |  |  |  |  |  |  |  |
| **RENAL MARKERS** |  |  |  |  |  |  |  |  |  |  |  |  |  |  |  |
| Creatinine |  |  |  |  |  |  |  |  |  |  |  |  |  |  |  |
| **BACTERIAL INFECTION MARKER** |  |  |  |  |  |  |  |  |  |  |  |  |  |  |  |
| Procalcitonin |  |  |  |  |  |  |  |  |  |  |  |  |  |  |  |

**Patient Medication Record:**

| Drug name | Route | Dose | Frequency | Duration (start & stop date) |
| --- | --- | --- | --- | --- |
|  |  |  |  |  |
|  |  |  |  |  |
|  |  |  |  |  |
|  |  |  |  |  |
|  |  |  |  |  |
|  |  |  |  |  |
|  |  |  |  |  |

**Therapeutic Regimen:**

| **Carbapenem Monotherapy:**  Meropenem  Imipenem/cilastatin  Ertapenem | **Carbapenem in combination with other antibiotics:**  Carbapenems with Penicillins  Carbapenems with Cephalosporin  Carbapenems with Quinolones  Carbapenems with Aminoglycosides  Carbapenems with colistin  Carbapenems with tetracyclines  Carbapenems with Vancomycin  Carbapenems with monoamine oxidase  Inhibitor (LZD) |
| --- | --- |

**Clinical Outcomes and Mortality Rate:**

| Hospital stay  ≥ 3- 10 days  v  11 – 15 days  16 – 21 days  ≥ 22 days | Treatment Duration  ≥ 3- 10 days  v  11 – 15 days  v  16 – 21 days  ≥ 22 days | End of Treatment  Successful  Cure & improvement  Failure  Patient death due to HAP  Patient death due to VAP  vvv  Death due to others  vvvv |
| --- | --- | --- |

**Drug related Problems:**

**Adverse effect**

vvvv

CNS (seizures)	CVS

vvvv

vvvv

Renal impairment	Dermatologic

vvvv

vvvv

Hepatic impairment	Genitourinary

vvvv

vvv

Gastro intestinal	Neuromuscular & skeletal

vvvv

vvvv

Hematologic	Respiratory

v vvvv

vvvv

Infection	local (inflammation at injection site)

vvv vvvv
