# Supplementary material for: Carbapenem-based versus non-carbapenem therapy for hospital-acquired and ventilator-associated pneumonia in a resource-limited setting: a retrospective cohort study of clinical outcomes
Source: J Pharm Pharm Sci. 2026 Sep 28;29:17336. doi: 10.3389/jpps.2026.17336 (PMC13654855; doi:10.3389/jpps.2026.17336)
Supplement: Supplementary file 2 [file Table1.docx]

## Table: Summary of Treatment Regimens by Group

| reatment Group | Pneumonia Type | n | Primary Agent(s) | Regimen Details (Dose, Frequency, Route) | Median Duration (Days, IQR) | Combination Therapy n (%) | Empirical n (%) | Definitive n (%) | Susceptible n (%) | Resistant n (%) |
| --- | --- | --- | --- | --- | --- | --- | --- | --- | --- | --- |
| **Carbapenem-based** | HAP | 38 | Meropenem ± Vancomycin ± Colistin | MERO: 0.5–1 g IV Q8H–Q24H;  VANC: 1–2 g IV Q12H–Q72H;  COL: 3–9 MU IV Q8H–Q24H | 8 (4–13) | 29 (76.3) | 31 (81.6) | 7 (18.4) | 22 (57.9) | 10 (26.3) |
| **Carbapenem-based** | VAP | 23 | Meropenem ± Vancomycin ± Colistin | MERO: 0.5–1 g IV Q8H–Q24H;  VANC: 1–2 g IV Q12H–Q72H;  COL: 3–9 MU IV Q8H–Q24H | 13 (7–19) | 21 (91.3) | 18 (78.3) | 5 (21.7) | 10 (43.5) | 8 (34.8) |
| **Non-carbapenem** | HAP | 24 | Piperacillin-tazobactam, Ceftazidime, Colistin, Levofloxacin, Teicoplanin, Tigecycline | PIP: 2.25–4.5 g IV Q6H–Q8H;  CEFT: 1–2 g IV Q8H–Q24H;  COL: 1.5–9 MU IV Q8H–Q12H;  LEVO: 750 mg IV/PO Q24H;  TEICO: 792 mg IV STAT/Q12H/Q72H;  TIGE: 50–100 mg IV Q12H | 8 (3–17) | 6 (25.0) | 18 (75.0) | 6 (25.0) | 14 (58.3) | 4 (16.7) |
| **Non-carbapenem** | VAP | 8 | Piperacillin-tazobactam, Ceftazidime, Colistin | PIP: 2.25–4.5 g IV Q6H–Q8H;  CEFT: 1–2 g IV Q8H–Q24H;  COL: 1.5–9 MU IV Q8H–Q12H | 14 (9–36) | 3 (37.5) | 5 (62.5) | 3 (37.5) | 4 (50.0) | 2 (25.0) |

*Abbreviations: HAP, hospital-acquired pneumonia; VAP, ventilator-associated pneumonia; IQR, interquartile range; MU, million units; MERO, meropenem; VANC, vancomycin; COL, colistin; PIP, piperacillin-tazobactam; CEFT, ceftazidime; LEVO, levofloxacin; TEICO, teicoplanin; TIGE, tigecycline. Dose ranges represent the minimum and maximum doses administered across patients in each group. '±' indicates that the agent was sometimes used in combination with other agents; not all patients received all listed agents.*
