# Supplementary material for: Carbapenem-based versus non-carbapenem therapy for hospital-acquired and ventilator-associated pneumonia in a resource-limited setting: a retrospective cohort study of clinical outcomes
Source: J Pharm Pharm Sci. 2026 Sep 28;29:17336. doi: 10.3389/jpps.2026.17336 (PMC13654855; doi:10.3389/jpps.2026.17336)
Supplement: Supplementary file 3 [file Table2.docx]

**Table: Subgroup Analysis of Carbapenem vs Non-Carbapenem Therapy on Mortality**

| Subgroup | Carbapenem (n/N, %) | Non-Carbapenem (n/N, %) | RR (95% CI) | p-value |
| --- | --- | --- | --- | --- |
| Critical | 26/47 (55.3) | 10/20 (50.0) | 1.11 (0.67-1.84) | 0.689 |
| Non-critical* | 1/14 (7.1) | 1/12 (8.3) | 0.86 (0.06-12.28) | 0.910 |
| ICU | 20/28 (71.4) | 7/13 (53.8) | 1.33 (0.76-2.31) | 0.269 |
| Non-ICU | 7/33 (21.2) | 4/19 (21.1) | 1.01 (0.34-3.02) | 0.989 |
| Diabetic | 10/23 (43.5) | 3/7 (42.9) | 1.01 (0.38-2.69) | 1.000 |
| Non-diabetic | 17/38 (44.7)) | 8/25 (32.0) | 1.40 (0.71-2.74) | 0.431 |

*Abbreviations: RR, risk ratio; CI, confidence interval; ICU, intensive care unit.*

Note: Wide confidence intervals in the non-critical subgroup are due to small sample size.
