# Supplementary material for: Carbapenem-based versus non-carbapenem therapy for hospital-acquired and ventilator-associated pneumonia in a resource-limited setting: a retrospective cohort study of clinical outcomes
Source: J Pharm Pharm Sci. 2026 Sep 28;29:17336. doi: 10.3389/jpps.2026.17336 (PMC13654855; doi:10.3389/jpps.2026.17336)
Supplement: Supplementary file 4 [file Table3.docx]

**Table: Pathogen-Specific Mortality Stratified by Pneumonia Type and Treatment**

| Subgroup | Pathogen Group | Died, n/N (%) |
| --- | --- | --- |
| **HAP (n=62)** |  |  |
|  | Acinetobacter group | 6/10 (60.0) |
|  | Pseudomonas group | 2/9 (22.2) |
|  | Other pathogens | 2/15 (13.3) |
|  | No growth | 9/28 (32.1) |
| **VAP (n=31)** |  |  |
|  | Acinetobacter group | 13/17 (76.5) |
|  | Pseudomonas group | 3/7 (42.9) |
|  | Other pathogens | 2/4 (50.0) |
|  | No growth | 1/3 (33.3) |
| **Carbapenem-treated (n=61)** |  |  |
|  | Acinetobacter group | 14/18 (77.8) |
|  | Pseudomonas group | 4/8 (50.0) |
|  | Other pathogens | 3/14 (21.4) |
|  | No growth | 6/21 (28.6) |
| **Non-carbapenem-treated (n=32)** |  |  |
|  | Acinetobacter group | 5/9 (55.6) |
|  | Pseudomonas group | 1/8 (12.5) |
|  | Other pathogens | 1/5 (20.0) |
|  | No growth | 4/10 (40.0) |

Abbreviations: HAP, hospital-acquired pneumonia; VAP, ventilator-associated pneumonia. Note: Pathogen groups are mutually exclusive. Polymicrobial infections were assigned using a hierarchy: Acinetobacter baumannii > Pseudomonas aeruginosa > other pathogens. Patients with no growth are classified separately.
