# Supplementary material for: Carbapenem-based versus non-carbapenem therapy for hospital-acquired and ventilator-associated pneumonia in a resource-limited setting: a retrospective cohort study of clinical outcomes
Source: J Pharm Pharm Sci. 2026 Sep 28;29:17336. doi: 10.3389/jpps.2026.17336 (PMC13654855; doi:10.3389/jpps.2026.17336)
Supplement: Supplementary file 5 [file Table4.docx]

**Table: Baseline Characteristics by Treatment Group**

| Characteristic | Carbapenem (n=61) | Non-Carbapenem (n=32) | p-value |
| --- | --- | --- | --- |
| **Age, mean (SD)** | 52.21 (17.48) | 50.28 (19.47) | 0.668* |
| **Gender, n (%)** |  |  | 0.853† |
| Male | 26 (42.6) | 13 (40.6) |  |
| Female | 35 (57.4) | 19 (59.4) |  |
| **Comorbidity, n (%)** |  |  | 0.085† |
| Diabetes | 23 (37.7) | 7 (21.9) |  |
| Other DM | 22 (36.1) | 9 (28.1) |  |
| No comorbidity | 16 (26.2) | 16 (50.0) |  |
| **Pneumonia Type, n (%)** |  |  | 0.217† |
| HAP | 38 (62.3) | 24 (75.0) |  |
| VAP | 23 (37.7) | 8 (25.0) |  |
| **Critical Illness, n (%)** |  |  | 0.137† |
| Critical | 47 (77.0) | 20 (62.5) |  |
| Non-critical | 14 (23.0) | 12 (37.5) |  |
| **Ward, n (%)** |  |  | 0.696† |
| MICU | 28 (45.9) | 13 (40.6) |  |
| ICU | 10 (16.4) | 4 (12.5) |  |
| HDU | 9 (14.8) | 4 (12.5) |  |
| CCU | 14 (23.0) | 11 (34.4) |  |
| General ward | 0 (0) | 0 (0) |  |

*Mann-Whitney U test.
†Chi-square test.
