# Supplementary material for: Carbapenem-based versus non-carbapenem therapy for hospital-acquired and ventilator-associated pneumonia in a resource-limited setting: a retrospective cohort study of clinical outcomes
Source: J Pharm Pharm Sci. 2026 Sep 28;29:17336. doi: 10.3389/jpps.2026.17336 (PMC13654855; doi:10.3389/jpps.2026.17336)
Supplement: Supplementary file 6 [file Table5.docx]

**Table: Detailed Polymicrobial Isolates in HAP and VAP**

| Polymicrobial Combination | HAP (n=62) | VAP (n=31) | Total |
| --- | --- | --- | --- |
| *A. baumannii* + *P. aeruginosa* | 1 | 3 | 4 |
| *A. baumannii* + *K. pneumoniae* | 0 | 2 | 2 |
| *A. baumannii* + *P. aeruginosa* + *K. pneumoniae* | 0 | 1 | 1 |
| *A. baumannii* + *S. aureus* | 2 | 2 | 4 |
| *P. aeruginosa* + MRSA + *E. coli* | 1 | 0 | 1 |
| *P. aeruginosa* + *S. aureus* | 1 | 1 | 2 |
| *A. baumannii* + *P. aeruginosa* + *S. aureus* | 0 | 2 | 2 |
| *A. baumannii* + *K. pneumoniae* + *S. aureus* | 0 | 1 | 1 |
| *P. aeruginosa* + *S. pneumoniae* + *H. influenzae* | 0 | 1 | 1 |
| **Total polymicrobial** | **5** | **13** | **18** |

*Note: Polymicrobial infections are defined as cultures yielding two or more organisms. Patients with polymicrobial infections were assigned to mutually exclusive pathogen groups using a hierarchy based on clinical significance: (1) Acinetobacter baumannii, (2) Pseudomonas aeruginosa, and (3) other pathogens*
