# Supplementary material for: Carbapenem-based versus non-carbapenem therapy for hospital-acquired and ventilator-associated pneumonia in a resource-limited setting: a retrospective cohort study of clinical outcomes
Source: J Pharm Pharm Sci. 2026 Sep 28;29:17336. doi: 10.3389/jpps.2026.17336 (PMC13654855; doi:10.3389/jpps.2026.17336)
Supplement: Supplementary file 7 [file Table6.docx]

**Table: Length of Stay by Survival Status**

| Group | Survival Status | n | Median (Days) | Q1 | Q3 | IQR | P values* |
| --- | --- | --- | --- | --- | --- | --- | --- |
| **HAP** | Died | 19 | 7.00 | 3.00 | 13.00 | 3.00 - 13.00 | 0.520 |
|  | Survived | 43 | 8.00 | 5.00 | 15.00 | 5.00 - 15.00 |  |
| **VAP** | Died | 19 | 10.00 | 4.00 | 18.00 | 4.00 - 18.00 | 0.109 |
|  | Survived | 12 | 19.50 | 10.50 | 30.50 | 10.50 - 30.50 |  |

*Mann-Whitney U test
